# Beyond Pharmaceuticals: Fit-for-Purpose New Approach Methodologies for Environmental Cardiotoxicity Testing

**DOI:** 10.14573/altex.2109131

**Published:** 2022-06-01

**Authors:** Mark C. Daley, Ulrike Mende, Bum-Rak Choi, Patrick D. McMullen, Kareen L. K. Coulombe

**Affiliations:** 1 Center for Biomedical Engineering, School of Engineering and Division of Biology and Medicine, Brown University, Providence, RI, USA;; 2 Cardiovascular Research Center, Cardiovascular Institute, Rhode Island Hospital and Warren Alpert Medical School of Brown University, Providence, RI, USA;; 3 ScitoVation, Durham, NC, USA

## Abstract

Environmental factors play a substantial role in determining cardiovascular health, but data informing the risks presented by environmental toxicants is insufficient. *In vitro* new approach methodologies (NAMs) offer a promising approach with which to address the limitations of traditional *in vivo* and *in vitro* assays for assessing cardiotoxicity. Driven largely by the needs of pharmaceutical toxicity testing, considerable progress in developing NAMs for cardiotoxicity analysis has already been made. As the scientific and regulatory interest in NAMs for environmental chemicals continues to grow, a thorough understanding of the unique features of environmental cardiotoxicants and their associated cardiotoxicities is needed. Here, we review the key characteristics of as well as important regulatory and biological considerations for fit-for-purpose NAMs for environmental cardiotoxicity. By emphasizing the challenges and opportunities presented by NAMs for environmental cardiotoxicity we hope to accelerate their development, acceptance, and application.

## 1 The need for improved cardiotoxicity evaluation of environmental chemicals

Environmental factors significantly affect global health outcomes, and cardiovascular disease (CVD), the leading cause of mortality worldwide, is no exception (Prüss-Ustün and Corvalán, 2006; Virani et al., 2020). An estimated 7–23% of CVD can be attributed to environmental factors such as air pollution, occupational hazards, and agricultural run-off (Prüss-Ustün and Corvalán, 2006), and a broad range of environmental chemicals is known to present cardiac-specific risk (Lind et al., 2021; Cosselman et al., 2015). Despite this, there is a shortage of knowledge regarding the cardiac-specific risks presented by environmental toxicants (Judson et al., 2009). This gap is in large part because current cardiotoxicity testing methods have a limited ability to predict structural, electrophysiological, and contractile cardiotoxicity independent of the underlying molecular mechanism.

### 1.1 Current cardiotoxicity testing

The major focus of recent advances in cardiotoxicity testing methods has been electrophysiological, largely due to the United States Food and Drug Administration’s (FDA) adoption of the International Council for Harmonisation of Technical Requirements for Pharmaceuticals for Human Use (ICH) guideline S7B in 2005 (Magdy et al., 2018; ICH, 2001, 2005). ICH S7B describes *in vivo* and *in vitro* models to detect delayed ventricular repolarization, a major risk factor of ventricular arrythmias (ICH, 2005). Developed in response to the rapid withdrawal of prenylamine, lidoflazine, and tordiline between 1988 and 1991 due to increased incidence of torsade de pointes, a deadly ventricular arrhythmia, it rapidly set the standard for pre-clinical evaluation of cardiotoxicity.

*In vivo* assessment of delayed repolarization under ICH S7B relies on measuring electrophysiological features identified via electrocardiogram, primarily the QT interval, defined as time between the start of the QRS complex and end of the T wave and a metric of the duration of ventricular depolarization and repolarization (ICH, 2005; Gintant et al., 2016). Prolongation of the QT interval in animal models is considered indicative of delayed repolarization and increased arrythmia risk in humans. However, the genetic homogeny of inbred rodents and inter-species physiological differences between rodents and human decrease the utility of animal studies for predicting human risk (Fischer et al., 2020; Gintant et al., 2016; Magdy et al., 2018; Olson et al., 2000). For example, disparities in the expression of ion channels between human and rodent cardiomyocytes result in different heart rates and action potential kinetics, leading to substantial inter-species differences in the effects of drugs and chemicals on QT prolongation (Mercola et al., 2013; Nerbonne et al., 2001; Sallam et al., 2015; Gintant et al., 2016; O’Hara and Rudy, 2012). These extrapolation challenges are especially relevant for apical endpoints that provide limited mechanistic insight (Gintant et al., 2016) for *in vivo* model systems that are already expensive to implement and have limited throughput. In addition, ethical concerns surround the use of animals in toxicology, and efforts to replace, reduce, and refine *in vivo* models in biomedical research have lessened their popularity (Törnqvist et al., 2014) and even resulted in the explicit ban of animal testing in some sectors, such as the European cosmetics industry (Fischer et al., 2020).

The predominant *in vitro* cardiotoxicity assay is the functional I_Kr_ assay, which is the *in vitro* portion of ICH S7B for detecting delayed repolarization (Gintant et al., 2016; Chen et al., 2016; ICH, 2005). Also known as the hERG assay, this screen examines changes in activity of the voltage-sensitive rapid delayed rectifier potassium channel, the primary facilitator of ventricular repolarization, whose pore-forming α subunit is encoded by the human ether-a-go-go related gene (*hERG*) (Priest et al., 2008). Under modern nomenclature, the α subunit is known as Kv11.1 and *hERG* as *KCNH2*. Blockade of this channel increases action potential duration and is associated with clinical QT prolongation, making the hERG assay a proxy for *in vivo* risk of ventricular fibrillation and torsade de pointes (Gintant et al., 2016; Priest et al., 2008). Unfortunately, this approach also has a limited predictive capacity for cardiotoxicity (Gintant, 2011). High-throughput versions of the assay, which rely on immortalized cell lines modified to express voltage-sensitive potassium channels, fail to reliably reflect *in vivo* behavior (Priest et al., 2008; Horvath et al., 2016). As a targeted, single-channel approach, this assay is also unable to identify effects on other ion channels critical for proper action potential kinetics (Gintant et al., 2016).

Neither the *in vivo* QT prolongation nor *in vitro* hERG assays defined in ICH S7B are designed to detect non-electrophysiological classes of cardiotoxicity (i.e., structural or contractile) that can arise individually or as a result of interacting mechanisms (Magdy et al., 2018; Gintant et al., 2016, 2019). Pre-clinical testing of non-electrophysiological cardiotoxicities is covered as a part of the core battery of *in vivo* assessments under the ICH S7A guidelines for general safety pharmacology. Cardiotoxicity is monitored by examining effects on blood pressure and heart rate in addition to the electrocardiogram *in vivo* with optional follow-ups for other functional characteristics like cardiac output and ventricular contractility in multiple animal species (ICH, 2001). *In vitro* assays that examine contractile or structural cardiotoxicity are not required, and early safety screening relies on optional target-based assays of known pathways of toxicity (PoTs) (Bowes et al., 2012). As a result, cardiotoxicity assessment of structural and contractile toxicities have substantial flexibility during pharmaceutical development, and many approaches and study species are applied (Guth, 2007; Sarazan et al., 2011). However, these methods possess many of the same challenges and drawbacks as *in vivo* studies and single-target *in vitro* assays performed for electrophysiological assessment (Fischer et al., 2020; Gintant et al., 2016; Magdy et al., 2018; Olson et al., 2000; Törnqvist et al., 2014).

These structural, contractile, and non-hERG-related electrophysiological cardiotoxicities are increasingly appreciated as significant hazards among environmental chemicals, occurring via multiple overlapping and often incompletely understood mechanisms even for well-studied toxicants (Lind et al., 2021; Cosselman et al., 2015). Exposure to heavy metals (e.g., arsenic, cadmium, chromium, mercury, and lead) has been associated with various non-electrophysiological cardiotoxicities such as oxidative stress, sarcomere disorganization, mitochondrial dysfunction, and calcium dysregulation in addition to increased arrhythmia risk (Sevim et al., 2020; Balali-Mood et al., 2021; Lind et al., 2021; Alissa and Ferns, 2011). Polycyclic aromatic hydrocarbons (PAHs), formed as byproducts of partial combustion and constituents of crude oil, have been associated with excitation-contraction coupling dysfunction and cardiac hypertrophy (Alhamdow et al., 2017; Marris et al., 2020). Polychlorinated biphenyls (PCBs), a category of industrial chemicals, have been linked to impaired left ventricular systolic and diastolic function (Lind et al., 2013) and heart failure (Åkesson et al., 2019). Mechanistic studies of PAH- and PCB-related cardiotoxicity suggest these effects arise from multiple aryl hydrocarbon receptor-dependent and -independent pathways (Incardona, 2017). Bisphenol A (BPA) is also a known cardiac toxicant, and exposure has been associated with increased risk of arrhythmias, cardiac dysfunction, and heart failure (Cai et al., 2020; Gao and Wang, 2014). Organochlorine, organophosphate, and carbamate pesticides are likewise known to cause contractile dysfunction and other cardiotoxicities in addition to electrophysiological effects (Georgiadis et al., 2018). Consequently, neither current *in vivo* nor *in vitro* approaches are well-suited for effective assessment of the full breadth of cardiac hazards presented by environmental toxicants.

### 1.2 New approach methodologies (NAMs)

There is a growing push for NAMs for human cardiotoxicity testing and safety assessment that address the challenges and limitations of current animal models and existing *in vitro* assays (Fischer et al., 2020; Parish et al., 2020; Zink et al., 2020). This new direction for toxicity assessment has largely been driven by the National Research Council report on Toxicology Testing in the 21^st^ Century, which provided a long-term vision for developing and adopting modern approaches for toxicity testing (NRC, 2007; Krewski et al., 2010). Similar initiatives have also been deployed in Europe (such as EU-ToxRisk) to promote NAM development in the European Union (Fischer et al., 2020; Daneshian et al., 2016). These initiatives aim to catalyze a new, more advanced paradigm that focuses on high-throughput, mechanistic analysis of the molecular PoTs associated with adverse outcomes through a range of enabling technologies (e.g., human stem cell culture, microfluidics, toxicogenomics, and *in silico* modeling) for specific decision-making needs (Kleensang et al., 2014; Parish et al., 2020; Ankley et al., 2010; Meek et al., 2014; Burnett et al., 2021b). NAMs that fulfill these criteria and are designed to meet specific biological and regulatory contexts are considered fit-for-purpose and are consistent with this vision of the future of toxicity testing (Parish et al., 2020).

This review examines the development of fit-for-purpose NAMs for the evaluation of the cardiotoxicity of environmental chemicals (i.e., assays of “environmental cardiotoxicity”). Specifically, we describe the unique elements of environmental toxicants and the features of fit-for-purpose NAMs that meet the needs of environmental safety assessment in the context of cardiac physiology. We hope to emphasize the challenges, current gaps, and need for further research and development of NAMs for environmental cardiotoxicity.

## 2 NAMs dedicated to environmental cardiotoxicity

While there is a pressing need to assess the cardiotoxic risks of an ever-increasing number of environmental chemicals (Judson et al., 2009), the majority of existing cardiotoxicity tests do not sufficiently account for the unique facets of environmental toxicants (Dix et al., 2007; Truskey, 2018).

### 2.1 Environmental toxicants possess more varied chemistries than pharmaceuticals

**Fig. 1:**
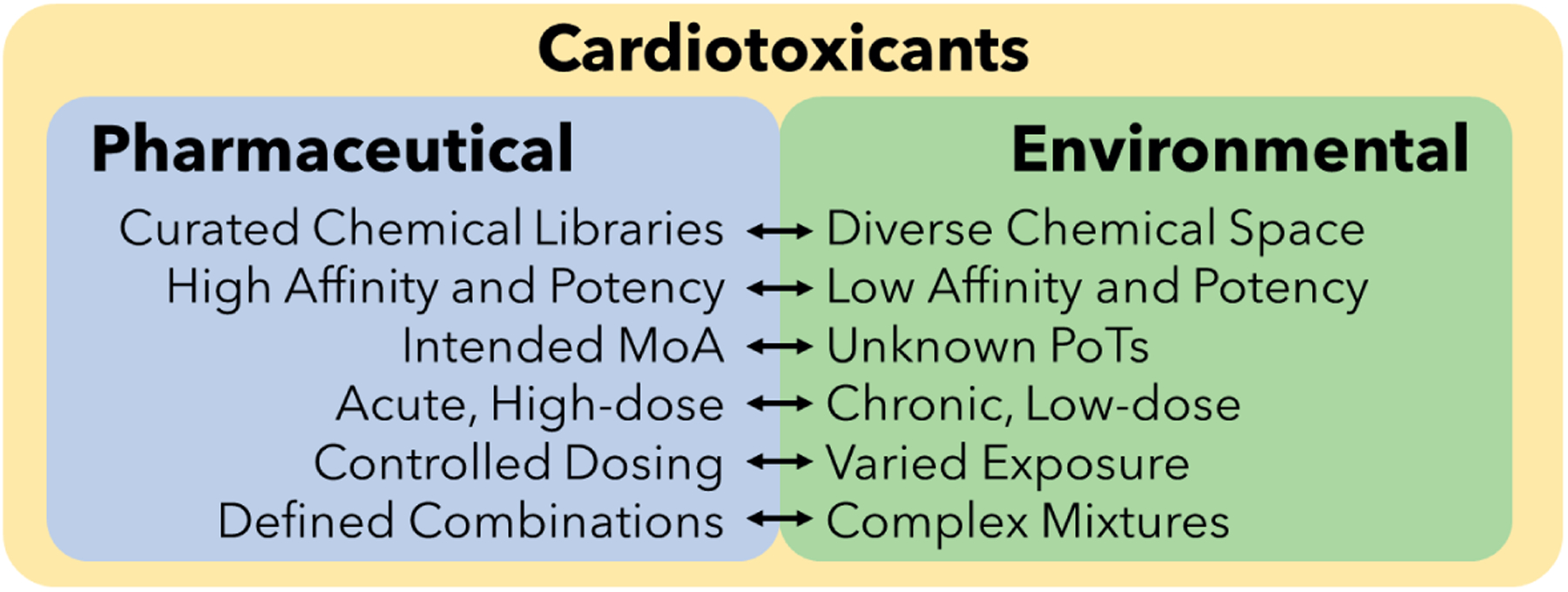
Comparison of the defining features of pharmaceutical compounds and environmental chemicals for cardiotoxicity testing Fit-for-purpose NAMs should account for critical differences in the chemical properties and exposure profiles of pharmaceutical compounds (left) and environmental chemicals (right). MoA, mechanism of action; NAMs, new approach methodologies; PoTs, pathways of toxicity

Most NAMs developed for cardiotoxicity have been optimized for pharmaceutical applications (Magdy et al., 2018; Gintant et al., 2019; Chen et al., 2016). Potential environmental toxicants, however, represent a much broader chemical space with less well-understood mechanisms of toxicity and lower potencies (Fig. 1) (Dix et al., 2007). This pharmaceutical focus means that current *in vitro* test methods were developed using chemical libraries curated to prioritize compounds for their likelihood to be pharmaceutical leads (e.g., having a low molecular weight and high water solubility) (Hann and Oprea, 2004; Lipinski et al., 1997). As a result, prospective pharmaceuticals undergoing toxicity tests represent a substantially homogenized subset of the physicochemical space available to modern chemists (Fig. 1, left) (Dix et al., 2007; Hann and Oprea, 2004). In contrast, environmental chemicals occupy a wider spectrum of chemistries because they represent compounds designed for more diverse applications (Fig. 1, right). This variety, in combination with the absence of selection for high-affinity biological activity, makes the needs of environmental cardiotoxicity testing substantially different from those of the pharmaceutical industry. The most useful NAMs for environmental cardiotoxicity will be able to detect cardiotoxicity even when arising from such low potency binding and activation of unanticipated PoTs.

### 2.2 Environmental toxicants have unique exposure profiles

Environmental toxicants are not only defined by their broad chemistries but also their distinct exposure profiles (Fig. 1). Common “far-field” exposure scenarios can be characterized by small chronic doses in complex mixtures (Krewski et al., 2010) with multiple routes of exposure such as inhalation via air pollution and ingestion via drinking water (Yang and Massey, 2019; Cosselman et al., 2015). These conditions differ substantially from the acutely administered single compounds of traditional cardiotoxicity testing. Moreover, the lower potencies of environmental toxicants make the ability to detect meaningful biological outcomes more difficult under chronic conditions (Dix et al., 2007). As such, NAMs designed for the detection of environmental cardiotoxicity will require approaches that compensate for these differences. This challenge can be met in part by NAMs that have improved sensitivity in detecting environmental cardiotoxicity. Incorporation of more diverse and holistic methods, such as experimental or computational *in vitro* to *in vivo* extrapolation (IVIVE), should enable a greater quantitative understanding of complex environmental exposures such as chronic inhalation. Methods that mimic and model longer-term exposures to environmental toxicants rather than acute biological effects will be those best suited for integration into NAMs for environmental cardiotoxicity.

### 2.3 Environmental exposures affect broad populations

Individuals exposed to environmental toxicants represent diverse populations in terms of their physiology, genetics, and cardiovascular health, each of which can compound the risk presented by otherwise sub-toxic environmental exposures, a scenario referred to as “hidden cardiotoxicity” (Frommeyer and Eckardt, 2016; Virani et al., 2020; Ferdinandy et al., 2019). NAMs for environmental cardiotoxicity will need to incorporate methods already being adopted for precision medicine to predict cardiotoxicity in individuals and populations with diverse genetic backgrounds (e.g., multiple human induced pluripotent stem cell (hiPSC) donors) and potential comorbidities (e.g., myocardial infarction) (Chen et al., 2016). Consideration of such risk factors will enhance the ability of NAMs for environmental cardiotoxicity to provide population-representative risk assessment.

### 2.4 Environmental toxicant properties drive effective NAM design

Specific attention to the unique aspects of environmental toxicity assessment should guide continued research and design of NAMs for cardiotoxicity analysis. Due to the diverse nature of environmental toxicants and a variety of regulatory decision points, it is unlikely that a single testing paradigm will be sufficient (Hartung et al., 2013). Rather, NAMs should be designed intentionally to complement both the biological (i.e., type of toxicity) and regulatory (i.e., type of decision) questions asked by stakeholders (Parish et al., 2020; Sauve-Ciencewicki et al., 2019). Reflection on their intended context of use will determine required accuracy and permittable limitations. In the following sections, we will discuss the factors that determine if a NAM is fit-for-purpose for assessing environmental cardiotoxic risk in real-world contexts.

## 3 Regulatory considerations for fit-for-purpose environmental cardiotoxicity evaluation

From a regulatory standpoint, toxicity tests can be broadly categorized by the information they provide as prioritization screens, hazard screens, and risk assessment platforms (Parish et al., 2020). Novel chemicals may require prioritization screening whereas chemicals already in the environment with suspected toxicity may need more detailed assessment. Effective NAMs for environmental cardiotoxicity will need to be cost-, time-, and resource-efficient, requiring optimization for their specific decision-making context or multiplexing to maximize the information available from a single assay. Examples of context-focused NAMs are provided below to exhibit highly tuned, fit-for-purpose platforms. The NAMs that are ultimately adopted will be those most predictive within their contexts of use, not necessarily the most technically complex or biologically complete. Thus, a thorough delineation of the intended application or applications should inform the necessary physiological features to be captured in an assay.

### 3.1 NAMs for environmental cardiotoxicity as prioritization screens

Toxicity tests that fall into the prioritization category are designed to generate an ordered list of potential toxicants that require additional evaluation. While they may not provide detailed mechanistic insight, broad assessment of relevant metrics is acceptable in early decision-making prior to substantial resource commitment. Examples of this type of cardiotoxicity analysis are already being developed for environmental toxicants (Sirenko et al., 2017), pharmaceutical chemicals (Sirenko et al., 2013; Sharma et al., 2017), and combinations of both (Krishna et al., 2020). These methods analyze cardiotoxicity data derived *in vitro* and aggregate dose-normalized metrics to calculate a safety or priority index, ranking compounds by relative risk. Even among diverse environmental toxicants, this technique can cluster chemicals by class (i.e., pesticides, flame retardants, and PAHs) and hazard type (Sirenko et al., 2017). This technique has also been used to identify major physicochemical properties associated with increased cardiotoxicity of related PCBs, identifying key structures and metabolites as risk factors to human health (Grimm et al., 2020). In the case of pharmaceuticals, which possess more fully characterized cardiotoxicity profiles, prioritization largely mirrors known cardiotoxicity (Sirenko et al., 2013; Sharma et al., 2017). For tyrosine kinase inhibitors, a subset of anti-cancer therapeutics with wide-ranging cardiovascular side effects, prioritization further discerns between inhibitor sub-classes (Sharma et al., 2017). These insights guided subsequent experiments that provided data on a novel mechanism of tyrosine kinase inhibitor-induced cardiotoxicity (Sharma et al., 2017). This method is particularly powerful when leveraging data generated from high-throughput screens, allowing cardiotoxicity ranking across a broad spectrum of compounds (Krishna et al., 2020). Application of machine learning approaches to a similar high-throughput targeted hERG screen has further demonstrated that prioritization screens can enable accurate quantitative structure-activity relationship predictions for a given PoT (Krishna et al., 2022). Expansion of such techniques to datasets generated from NAMs that more broadly reproduce cardiac physiology will permit rapid prioritization of cardiotoxicity from multiple mechanisms based on chemical structure. Such high-level prioritization using next generation NAMs for environmental cardiotoxicity will ultimately enable efficient risk mitigation and biological discovery.

### 3.2 NAMs for environmental cardiotoxicity as hazard screens

While offering broad insight, prioritization screens themselves are unlikely to provide the detail needed to identify individual PoTs (Parish et al., 2020). Hazard screens fill this niche, confirming cardiotoxicity identified during prioritization, providing additional mechanistic insight, and predicting harmful concentrations. Existing platforms are predominantly designed to examine structural (Archer et al., 2018; Polonchuk et al., 2017), electrophysiological (Ando et al., 2017; Blinova et al., 2017, 2018; da Rocha et al., 2020; Grimm et al., 2015, 2018; Guo et al., 2013; Ravenscroft et al., 2016; Pfeiffer-Kaushik et al., 2019; Kofron et al., 2021), or contractile (Agarwal et al., 2013; Feric et al., 2019; Huebsch et al., 2016; Mathur et al., 2015; Pointon et al., 2017; Skardal et al., 2017; Nunes et al., 2013; Lee et al., 2015) changes separately. For example, characterization of the structural cardiotoxicity experienced by spheroids *in vitro* could classify toxicants by the primary mechanism of toxicity (i.e., cell viability, mitochondrial toxicity, disruption of the endoplasmic reticulum) (Archer et al., 2018). Similarly, multi-parameter analysis of changes in the field potential shape of hiPSC-CMs using microelectrode arrays has been able to categorize compounds by the ion channel they disrupt and their proarrhythmic potential (Clements and Thomas, 2014). Finally, a contractile cardiotoxicity model leveraging high-throughput imaging of cardiac spheroids effectively distinguished inotropic and non-inotropic compounds during acute exposure (Pointon et al., 2017). While increasing the complexity, expense, and duration of testing, integrated systems that combine multiple hazard screens may be required to capture the full range of cardiac PoTs (Hartung et al., 2013). Models that examine multiple classes of cardiotoxicity concurrently could allow identification of the predominant toxicity (Beauchamp et al., 2015, 2020; Burridge et al., 2016; Clements et al., 2015; Forsythe et al., 2018; Schaaf et al., 2011; Chaudhari et al., 2018).

### 3.3 NAMs for environmental cardiotoxicity as risk assessment platforms

Hazard screens are ultimately limited in their ability to inform policymakers of real environmental risk because these screens do not incorporate data on actual toxicant exposures (i.e., concentration and duration), instead examining acute effects at relatively high doses (Krewski et al., 2010). NAMs that seek to mimic dosage, timing, and complex systems interactions will be better suited to assess risk by linking observations to real-world exposures and outcomes via IVIVE through experimental and computational methods (Parish et al., 2020; Wetmore, 2015; Bell et al., 2018).

The most straightforward approach is to manually apply representative, chronic dosing regimens to existing *in vitro* cardiotoxicity models. Chronic exposure of hiPSC-CMs to physiologically relevant doses of ethanol over five days has demonstrated dose- and time-dependent effects on cardiomyocyte viability, calcium transients, and gene expression (Rampoldi et al., 2019). This has also been demonstrated employing physiologically based pharmacokinetic (PBPK) modeling and hiPSC-CM spheroids to examine doxorubicin cardiotoxicity *in vitro* with time-dependent concentrations that mimic *in vivo* clearance over two weeks (Verheijen et al., 2018). This study observed that spheroids exposed to therapeutic regimens mirroring *in vivo* pharmacokinetics show phenotypic responses that mimic the chronic cardiotoxicity observed in cancer patients, highlighting the importance of realistic exposure profiles in chronic toxicity testing. The reverse process is also possible. Rather than using pharmacokinetic modeling to predetermine dosing conditions, computational IVIVE models can be applied to predict the real-world exposures that result in tissue-level concentrations equivalent to those determined to be toxic within *in vitro* assays (Wetmore et al., 2013).

Approaches that employ more complex *in vitro* systems may further advance IVIVE by incorporating critical components of toxicant absorption, distribution, metabolism, and elimination. Microphysiological systems (MPSs) can potentially be leveraged to model pharmacokinetics and multi-organ interactions *in vitro* (Truskey, 2018). In emerging approaches, cardiac cells cultured in MPSs that incorporate functional liver cells demonstrate metabolite-triggered cardiotoxicities that are otherwise absent (Skardal et al., 2020; Lee-Montiel et al., 2021). While there are many advantages granted by this increase in assay complexity, incorporation of such techniques will inherently be need- and context-dependent.

### 3.4 NAMs for environmental cardiotoxicity for multiplexed decision-making

Multiplexed assays that provide information about multiple decision-making contexts offer one option with which to increase efficiency. The examination of multiple, functional outcomes in combination with supplemental computational approaches has demonstrated the ability to simultaneously identify chemical hazards and provide population-level risk assessment for hundreds of chemicals (Blanchette et al., 2020; Burnett et al., 2021a). Such large-scale approaches can provide additional insight at multiple scales due to their ability to better characterize the variability in human cardiotoxic response while minimizing experimental burden. The scope of these assays, however, presents challenges surrounding their increased experimental and analytical complexity (Chiu and Rusyn, 2018) and can require numerous or carefully selected donor cell lines to maximize coverage of population effects (Blanchette et al., 2022). Should such hurdles be overcome, multiplexing may provide substantial advantages in understanding and predicting environmental cardiotoxicity, and incorporation of these tools should be considered as a method to improve the efficiency of large-scale testing of diverse environmental chemicals.

## 4 Biological considerations of fit-for-purpose environmental cardiotoxicity evaluation

**Fig. 2:**
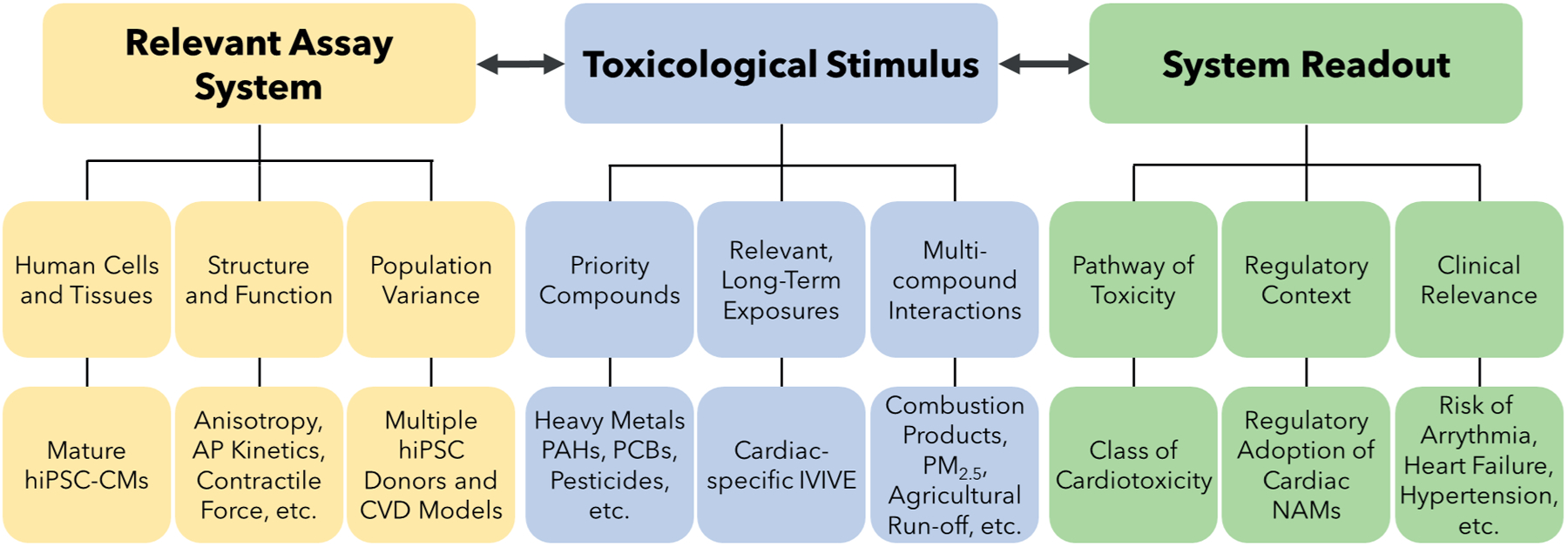
The chain of translatability for NAMs for environmental cardiotoxicity testing Effective NAMs should include (left) a biologically relevant assay system, (center) context-appropriate stimuli, and (right) actionable system readout. Underlying general and cardiac-specific criteria for each link in the chain are listed below their respective category (top and bottom, respectively). CVD, cardiovascular disease; hiPSC-CMs, human induced pluripotent stem cell-derived cardiomyocytes; IVIVE, *in vitro* to *in vivo* extrapolation; NAMs, new approach methodologies; PAHs, polyaromatic hydrocarbons; PCBs, polychlorinated biphenyls; PM_2.5_, fine particulate matter

From a biological standpoint, NAMs for cardiotoxicity assessment are defined by the pathophysiological processes they attempt to mimic and measure, including specific cell toxicities, whole organ responses, and population-level variations. To ensure a system sufficiently models this underlying physiology, effective NAMs establish a complete chain of translatability (Moffat et al., 2017), alternatively known as the rule of three (Vincent et al., 2015), consisting of a relevant assay system, toxicological stimulus, and system readout (Fig. 2). The NAMs that most closely resemble healthy or diseased cardiac physiology, toxicant exposure, and provide information directly relevant to health outcomes are the most likely to accurately predict cardiotoxicity. To establish this chain of translatability in NAMs for environmental cardiotoxicity, the unique features of environmental toxicants must be emphasized in each individual link.

### 4.1 Fit-for-purpose NAMs recapitulate cardiac physiology

**Fig. 3:**
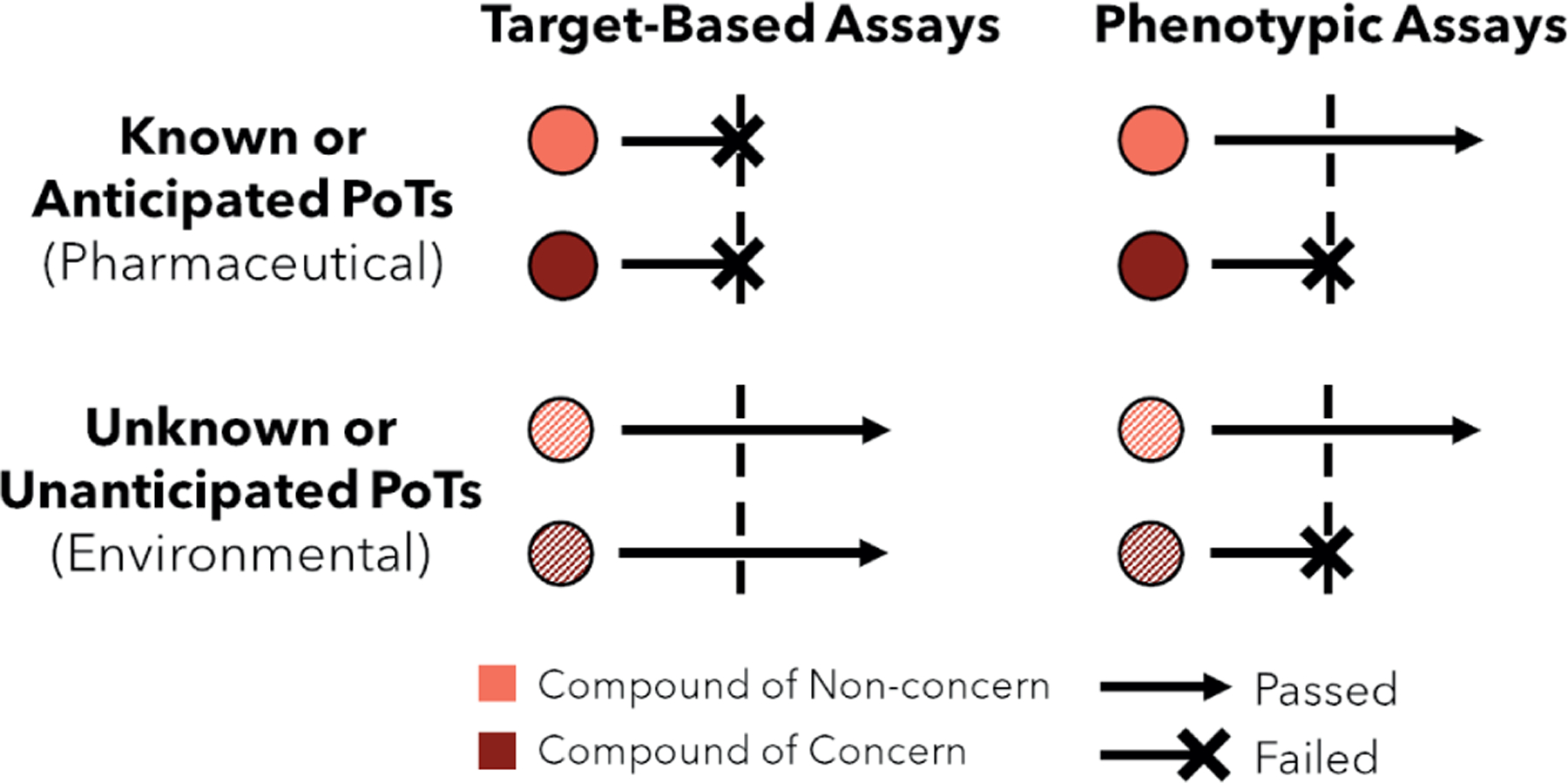
Comparison of traditional target-based and novel phenotypic *in vitro* assays in detecting known and unknown mechanisms of cardiotoxicity (Left) Target-based assays (e.g., hERG assay) isolate the effects of a single molecular target, allowing more sensitive detection of compounds with low-dose cardiotoxicity involving that PoT, but with a higher risk of false positives. (Right) Phenotypic assays (e.g., spheroid or microtissue, EHT, or MPS) that better mimic human cardiac physiology can detect toxicity that results from unknown or unanticipated PoTs from environmental cardiotoxicants but can possess reduced sensitivity to specific PoTs compared to target-based assays. EHT, engineered heart tissue; hERG, human ether-a-go-go related gene; MPS, microphysiological system; PoT, pathway of toxicity

The assay system is the most prominent aspect of NAM design and has been examined extensively in the context of pharmaceutical cardiotoxicity (Chen et al., 2016; Magdy et al., 2018; Gintant et al., 2019). As a result, significant progress has been made in developing relevant assay systems that mimic cardiac biology *in vitro* (Fig. 2). A large portion of this progress has been made by applying phenotypic methods that are intended to mimic biological systems in a target-agnostic manner (Moffat et al., 2017). Target-agnosticism allows phenotypic methods to be more effective in the discovery of first-in-class small molecules compared to target-based methods (Swinney, 2013; Swinney and Anthony, 2011), a scenario analogous to the identification of environmental toxicity from unknown mechanisms. Contrastingly, current target-based assays are sensitive to low-dose effects for known PoTs but are unlikely to detect even severe cardiotoxicity arising from pathways and mechanisms that are not explicitly considered in their design (Fig. 3). By circumventing the limitations of focused, target-centered assays, physiologically based NAMs provide a substantial opportunity for tackling the diverse chemical space and PoTs presented by environmental toxicants.

#### 4.1.1 Distinct features of cardiac physiology are reproduced by distinct NAM designs

Current systems range from two-dimensional cardiomyocyte culture and co-culture to three-dimensional spheroids or microtissues, engineered heart tissues (EHTs), and cardiac-specific MPSs (hearts-on-a-chip), each with their respective advantages and disadvantages (Tab. 1) (Gintant et al., 2019).

Two-dimensional monolayer systems are attractive due to their simplicity and ready integration into existing high-throughput screens including automated patch clamping, multi-electrode arrays, cellular impedance measurement, motion field imaging, and calcium imaging (Takasuna et al., 2017; Berg et al., 2014). However, two-dimensional cell culture is known to have a significant negative impact on many facets of cardiomyocyte phenotype (e.g., gene expression, metabolism, and contraction force) (Baker and Chen, 2012; Ahmed et al., 2020). Neonatal rat cardiomyocytes grown in three-dimensional aggregates display increased sensitivity to treatment with triiodothyronine (T3) and less fetal-like gene expression (Akins Jr et al., 2010). For hiPSC-CMs, culture in three-dimensional EHTs results in increased mitochondrial mass and oxidative phosphorylation compared to two-dimensional culture (Ulmer et al., 2018). As a result, there is an increasing interest in applying these techniques in models of cardiotoxicity.

Cardiac spheroids and microtissues, self-assembling aggregates of cardiac cells, can be easily created using relatively few cells and incorporated into multi-well platforms for image-based analysis using existing testing pipelines (Tab. 1) (Zuppinger, 2019; Meyer et al., 2019). However, they have limited utility for direct contractile force measurements because they lack the necessary mechanical loading and require sample pooling for some molecular analyses (Zuppinger, 2019; Meyer et al., 2019). By better modeling cardiac physiology while maintaining throughput, 3D spheroid models have demonstrated the potential for enhanced prediction of cardiotoxicity. In a comparison to hiPSC-CM monolayers, spheroids were shown to better detect the structural cardiotoxicity of 29 drugs previously approved by the FDA (Archer et al., 2018).

EHTs are created via the combination of cells and a biomaterial scaffold that is shaped by a variety of biomanufacturing methods (Bajaj et al., 2014; Mironov et al., 2009). This format provides anisotropy through physical geometrical constraints, offers microenvironmental control via biomaterial selection, and permits both direct (e.g., via force transducers) and indirect (e.g., recording post deflection) contractile force measurements (Tab. 1) (Zuppinger, 2019; Meyer et al., 2019). These features, along with maturation via electrical stimulation, have allowed the development of EHT-based assays that mirror *in vivo* concentration-dependent effects of compounds with known impacts on cardiac contractility (Feric et al., 2019). Their larger size, however, requires additional cells, limits tissue homogeneity as well as nutrient diffusion, and is generally more labor-intensive than spheroid- or microtissue-based models (Zuppinger, 2019).

MPSs apply microfabrication and microfluidic techniques to create *in vitro* systems that can mimic *in vivo* spatial arrangements within and between organs (Truskey, 2018). These systems enable the study of transport phenomena while on-chip designs permit ready integration into multi-organ models. Cardiac-only chips can provide cardiotoxicity testing platforms (Agarwal et al., 2013; Mathur et al., 2015) while the incorporation of other organ models, such as the liver, has been used to predict metabolite toxicity (Lee-Montiel et al., 2021; Skardal et al., 2020). However, the complexity of these systems, especially when integrating multiple organ systems, can limit throughput.

No single approach will fully capture the complexity of cardiac physiology. Already, recombination of existing approaches has occurred, resulting in spheroid-based MPSs (Skardal et al., 2017, 2020) as well as scaffold-free EHTs made from bio-printed spheroids (Ong et al., 2017). Further innovation of NAM designs is expected as techniques are further refined and recombined.

#### 4.1.2 hiPSC-derived cardiac cells enable individual- and population-level insight

Underlying the advances described above is a reliance on hiPSC-CMs and other hiPSC-derived cardiac cells. The lineages available to researchers now include multiple sub-types of cardiomyocytes (e.g., ventricular, atrial, and nodal) and non-cardiomyocytes (e.g., epicardial, endocardial, and fibroblast cells) (Protze et al., 2019; Mikryukov et al., 2021; Witty et al., 2014). Human cardiac cells overcome the fundamental physiological mismatch of animal models and other cell lines in structural, electrophysiological, and contractile cardiotoxicity (Magdy et al., 2018; Chen et al., 2016; Burnett et al., 2021b). This advantage arises because hiPSC-derived cells can capture aspects of human cardiac physiology better than non-human approaches due to species-specific gene and protein expression patterns. This is particularly true as continued improvements are made to current differentiation techniques that accelerate their maturation and enhance their purity *in vitro* (Zhao et al., 2020; Scuderi and Butcher, 2017; Gomez-Garcia et al., 2021; Schmid et al., 2021; Protze et al., 2019). For instance, the role of metabolic changes in driving cardiomyocyte maturity is increasingly recognized (Garbern and Lee, 2021). Culturing ventricular hiPSC-CMs in conditions that mimic the *in vivo* metabolic environment has been demonstrated to elicit significant improvements in sarcomere organization, force production, and calcium handling (Feyen et al., 2020). This technique has also shown that matured hiPSC-CMs improve the fidelity of *in vitro* disease modeling by capturing phenotypes not seen with previous methods (Feyen et al., 2020). Ramped electrical stimulation of EHTs has further been shown to improve the myofibril structure, calcium handling, and electrophysiological properties of cardiomyocytes *in vitro*, leading to concentration-responses predictive of known drug effects (Nunes et al., 2013; Feric et al., 2019). Results such as these emphasize that advances in hiPSC differentiation methods will benefit all aspects of cardiac physiology and continue to improve the sensitivity and accuracy of NAMs.

Population-level risk can also be elucidated due to the ability to readily derive hiPSCs from numerous individuals (Chen et al., 2016; Magdy et al., 2018). Early work characterizing differences in hiPSC-CMs derived from 27 individual donors via calcium flux analysis and high-content imaging showed reproducible inter-individual variability both at baseline and in response to cardiotoxic drugs (Grimm et al., 2018). This same approach, in combination with *in silico* pharmacodynamic modeling, was able to predict corrected QT prolongation of > 10 ms similarly to clinically standard thorough QT studies for arrhythmia risk (Blanchette et al., 2019). This work has been expanded to include 43 donors and over 100 compounds, including environmental toxicants, food constituents, and industrial chemicals, further establishing hiPSC-CMs as suitable for population-level environmental cardiotoxicity analysis (Burnett et al., 2019). More recently, this approach has been combined with *in silico* modelling to quantify toxicodynamic variability (Blanchette et al., 2020); it has also demonstrated feasibility when applied to an even broader range of over 1,000 chemicals in hiPSC-CMs from just five donors (Burnett et al., 2021a).

Healthy cardiac physiology, however, is not representative of a significant portion of the population due to the prevalence of CVD (Virani et al., 2020). Current *in vivo* and *in vitro* cardiotoxicity models typically mimic non-disease states, ignoring the compounding risk of underlying CVD (Ferdinandy et al., 2019). The use of hiPSC-CMs has enabled the study of a range of cardiac pathologies *in vitro*, particularly those that arise from monogenic mutations (e.g., long QT syndrome). This is because assay development for these conditions is more straightforward than for other, complex forms of CVD since the underlying etiology can be captured with hiPSC-CMs derived from donors with the condition or via modification of a single gene in an established hiPSC line (Chen et al., 2016; Mercola et al., 2013).

Models of more complex diseases and related changes in cardiotoxicity risk have already begun to be developed. A spheroid model of myocardial infarction that captured organotypic oxygen gradients *in vitro* has been shown to have increased sensitivity to doxorubicin toxicity compared to control spheroids (Richards et al., 2020). hiPSC-CMs derived from breast cancer patients that experienced doxorubicin-induced toxicity have also been demonstrated to possess increased susceptibility to doxorubicin compared to hiPSC-CMs from patients that had not experienced doxorubicin-induced toxicity (Burridge et al., 2016). This work illustrates the potential of NAMs to recapitulate complex risk factors of cardiotoxicity *in vitro*. Studies such as these suggest a role of NAMs in examining patient populations that would otherwise remain unstudied in current animal models or *in vitro* assays. Expansion of such analyses will be critical in addressing the risk of hidden environmental cardiotoxicity.

### 4.2 Fit-for-purpose NAMs incorporate real-world exposure conditions as toxicological stimuli

Incorporation of exposure conditions that more adequately represent toxicological stimuli relevant for environmental cardiotoxicity is a critical goal for fit-for-purpose NAMs (Fig. 2). Real-world environmental hazards involve multiple, long-term exposures, and the acute conditions commonly used in *in vitro* toxicity testing do not recapitulate these scenarios (Krewski et al., 2010). NAMs that integrate these factors will be better positioned to assess the risk of cardiotoxicity in actual populations, although challenges persist. NAMs utilizing lower exposures on the scale of weeks will need to remain viable while preserving the ability to detect subtle changes in cardiac function that reflect PoTs characteristic of environmental cardiotoxicity. Progress has already been made in developing NAMs that meet these goals. Application of low doses of doxorubicin to *in vitro* models over two weeks has been shown to activate alternate PoTs compared to high doses that elicit acute toxicity (Verheijen et al., 2018), and another recent example detected acute BPA-induced arrhythmogenesis in cardiac microtissues at the physiologically relevant dose of 1 nM via high-speed optical mapping (Kofron et al., 2021).

Cardiotoxicity risk assessment is further complicated by exposures to complex mixtures with potential complementary cardiotoxicity as well as substances with unknown or variable composition, complex reaction products, and biological materials (UVCBs) (Krewski et al., 2010). The chemical composition of exposures arising from aqueous (e.g., pesticide run-off or water disinfection by-products) or airborne (e.g., petroleum exhaust or volatile organic chemicals) sources vary both in time and space, and individual risk of these exposures is expected to vary significantly (Krewski et al., 2010; Pauluhn, 2005). In contrast, typical co-exposure toxicity assessments make simplifying assumptions regarding mixtures by treating them as single toxicants with identical PoTs (i.e., dose addition) or independent PoTs (i.e., response addition) (Teuschler et al., 2002). Mixtures and UVCBs, however, may invoke more complicated dynamics that may be impractical to reconstruct from information regarding individual exposures. Recent work applying NAMs to examine both individual environmental chemicals and complex mixtures directly suggests that these assumptions do not fully reflect risk (Hsieh et al., 2021). Fortunately, NAMs provide a feasible approach for examining these interactions. Two-dimensional models employing hiPSC-CMs have shown concentration dependent effects to gas oil extracts (Grimm et al., 2015), simple drug mixtures (Blinova et al., 2017), and the constituents of energy drinks (Luo et al., 2021). Cardiotoxic analysis of ground soil samples has further demonstrated the ability of NAMs to correlate *in vitro* risk assessment with the spatial distribution of contaminants to identify areas of concern after site contamination (Chen et al., 2021).

While these examples are promising, extrapolation of *in vitro* data of complex mixtures and UVCBs to equivalent whole-body exposures remains an outstanding challenge. A tiered IVIVE framework would allow integration of available data on absorption, distribution, metabolism, and elimination of mixture components (Wambaugh et al., 2015). Where gaps in data exist, computational approaches (e.g., quantitative structure-activity relationship models) can be used to inform dosimetry models. As advances in sensitivity, throughput, and IVIVE are made, NAMs are expected to enable a more complete understanding of the environmental cardiotoxicity of complex chemical environments and long-term exposures.

### 4.3 Fit-for-purpose NAMs provide actionable system readouts

*In vitro* observations and *in vivo* cardiotoxicity are best connected by an evaluation platform with an easily interpretable and clinically relevant readout, the final link in the chain of translatability (Fig. 2). The current standards are *in vivo* QT measurement via electrocardiogram, employed for its non-invasive collection and direct clinical applicability, and the *in vitro* hERG assay, leveraged for its simplicity and throughput, with each possessing well-established methodologies and standardized result interpretation (Gintant et al., 2016). Despite these advantages, however, both possess a disconnect between experimental readout and clinical outcomes (Frommeyer and Eckardt, 2016; Gintant et al., 2016). QT prolongation by itself is an imperfect surrogate for human arrhythmogenic risk due to multi-ion channel interactions that can compensate for extended repolarization times. For example, the drug ranolazine causes QT prolongation but is not proarrhythmic due to simultaneous blockade of late inward sodium currents (Gintant et al., 2016; Wu et al., 2009). The hERG assay similarly exemplifies such a mismatch because the single-channel test fails to account for these same compensatory effects on other ion currents (Gintant et al., 2016; Frommeyer and Eckardt, 2016). Verapamil, a known hERG inhibitor, does not elicit QT prolongation or arrhythmogenic risk due to concurrent blockade of inward calcium currents (Gintant et al., 2016; Zhang et al., 1999). In addition, most current methods are limited to electrophysiological readouts and do not address contractile or structural changes.

The need for broader *in vitro* endpoints that can be linked to *in vivo* manifestations of toxicity is already being addressed by NAMs that detect major classes of cardiotoxicity via simple, high-throughput readouts (Chen et al., 2016; Magdy et al., 2018). These include methods to measure structural (ATP activity, mitochondrial integrity, cell morphology), electrophysiological (micro-electrode arrays, voltage-sensitive dyes, calcium-sensitive dyes), and contractile (displacement tracking, post deflection) cardiotoxicity that can be effectively linked to clinical effects such as cardiomyocyte viability, arrythmia generation, and reduced ejection fraction, respectively. The increased biological complexity of NAMs has also increased interest in higher content “omics-level” data (Moffat et al., 2017; Pauluhn, 2005; Teuschler et al., 2002). While more difficult to interpret, omics-level data can provide holistic and detailed information regarding interacting PoTs of environmental toxicants and suggest omics-level signatures of cardiotoxicity liabilities (Burnett et al., 2021b). For instance, transcriptomic analysis of hiPSC-CMs exposed to doxorubicin and other anthracyclines, chemotherapeutics with well-known cardiotoxic side effects, have suggested a common set of deregulated genes that appears prior to the onset of other cytotoxicity markers for anthracycline-induced cardiotoxicity (Chaudhari et al., 2016). Ultimately, the type, number, and complexity of NAM readouts will be dictated by their intended context of use.

In conjunction with the clinical relevance of a readout is the need to consider how NAMs are to be validated and adopted by the wider research and regulatory communities (Parish et al., 2020; Marx et al., 2020). This is especially important when NAMs have not replaced older methods and are instead utilized in parallel (Krewski et al., 2010). Moreover, validation of *in vitro* NAMs has necessarily included comparison to *in vivo* pre-clinical animal data, which do not necessarily predict known human cardiotoxicity (Olson et al., 2000; ICCVAM, 2018; Ingber, 2020). Thus, regulators are shifting towards a model of integrated NAM validation that relies on empirical evidence to demonstrate human cardiac physiology and toxicity based on intended context of use and mechanistic relevance (Parish et al., 2020; ICCVAM, 2018; Ingber, 2020). It is this validation of predictive capacity in humans that will complete the biological chain of translatability for environmental cardiotoxicity testing.

## 5 Conclusion

A more complete understanding of how environmental toxicants influence human cardiovascular health and function will be essential to evaluating real-world risk. Substantial progress has been made in the past decade in developing NAMs for cardiotoxicity, but most efforts have focused on evaluating pharmaceutical, not environmental, compounds. Such advances lay a considerable foundation from which to build, with advances in hiPSC-CM-based approaches providing necessary physiological relevance for accurately capturing impacts on human cardiovascular health. To fully capitalize on this momentum and develop NAMs fit for assessing the cardiotoxicity of environmental compounds, a more deliberate consideration of their diverse chemical properties and distinct exposure conditions will be needed. Ultimately, the regulatory (i.e., prioritization, hazard screening, risk assessment) and biological (i.e., relevant physiology, realistic exposures, interpretable readouts) contexts of NAMs for environmental cardiotoxicity should align with their intended purpose. A robust suite of NAMs for cardiotoxicity will provide the tools necessary to realize the National Research Council’s vision for 21^st^ century toxicity testing for environmental cardiotoxicity.

## Figures and Tables

**Tab. 1:** Advantages and disadvantages of traditional target-based and phenotypic *in vitro* assays for cardiotoxicity testing Both traditional target-based monolayer assays (e.g., the hERG assay) as well as phenotypic *in vitro* assays employing hiPSC-CMs fulfill distinct niches in cardiotoxicity assessment.

	Model type	Advantages	Disadvantages
**Target-based**	Target-based monolayers (e.g., hERG assay)	Simple Sensitive Regulatory precedent	Non-human Frequent false positives Single-mechanism focus
**Phenotypic**	hiPSC-CM monolayers	Human Relatively simple 2D systems	Single cell type Low sensitivity metrics
	Microtissues (spheroids)	Human Minimal cells required Self-assembled Easy-to-image High sensitivity metrics	Isotropic Indirect force measurements Pooled molecular analysis
	Engineered heart tissues	Human Microenvironmental control Anisotropic tissues More direct force measurement	Larger size Limited homogeneity Limited nutrient diffusion
	Microphysiological systems (organ-on-a-chip)	Human Multi-organ capabilities Spatial control Fluid flow Easy-to-image	Labor intensive Specialized microfabrication

hERG, human ether-a-go-go related gene; PoT, pathway of toxicity; hiPSC-CMs, human induced pluripotent stem cell-derived cardiomyocytes

## Data Availability

No datasets were analyzed or generated as part of this manuscript.
